# Laser-Based Single-Axon Transection for High-Content Axon Injury and Regeneration Studies

**DOI:** 10.1371/journal.pone.0026832

**Published:** 2011-11-02

**Authors:** Darío Kunik, Carolyne Dion, Tsuneyuki Ozaki, Leonard A. Levin, Santiago Costantino

**Affiliations:** 1 Centre de Recherche de l'Hôpital Maisonneuve-Rosemont, Montréal, Quebec, Canada; 2 Institut de Génie Biomédical, Université de Montréal, Montréal, Quebec, Canada; 3 Energie Materiaux et Communications, Institut National de la Recherche Scientifique, Quebec City, Quebec, Canada; 4 Département d'Ophtalmologie Université de Montréal, Montréal, Quebec, Canada; 5 Department of Ophthalmology and Visual Sciences, University of Wisconsin, Madison, Wisconsin, United States of America; The University of Akron, United States of America

## Abstract

The investigation of the regenerative response of the neurons to axonal injury is essential to the development of new axoprotective therapies. Here we study the retinal neuronal RGC-5 cell line after laser transection, demonstrating that the ability of these cells to initiate a regenerative response correlates with axon length and cell motility after injury. We show that low energy picosecond laser pulses can achieve transection of unlabeled single axons *in vitro* and precisely induce damage with micron precision. We established the conditions to achieve axon transection, and characterized RGC-5 axon regeneration and cell body response using time-lapse microscopy. We developed an algorithm to analyze cell trajectories and established correlations between cell motility after injury, axon length, and the initiation of the regeneration response. The characterization of the motile response of axotomized RGC-5 cells showed that cells that were capable of repair or regrowth of damaged axons migrated more slowly than cells that could not. Moreover, we established that RGC-5 cells with long axons could not recover their injured axons, and such cells were much more motile. The platform we describe allows highly controlled axonal damage with subcellular resolution and the performance of high-content screening in cell cultures.

## Introduction

Significant effort has been devoted to elucidating the pathways that lead to neuronal dysfunction and death in diseases as disparate as Alzheimers disease, trauma, multiple sclerosis, peripheral neuropathies, and glaucoma [1]–[4]. In recent years, it has become evident that cell bodies (somas) and axons follow divergent degeneration pathways, with axonal injury or degeneration often preceding cell death [1], [5], [6]. After injury, isolated axons undergo Wallerian and retrograde degeneration, which are active axon degeneration processes independent of apoptosis and relevant to different diseases [3], [7] Although there is an excellent understanding of soma death programs and multiple methods for inhibiting soma death are available the pathways for axon degeneration and preventing axon loss are as yet poorly understood. Improved techniques for studying the responses of somas and axons to injury would therefore help in the development of therapies for these otherwise irreversible neuronal diseases.

Laser-based technologies have been shown to be powerful yet precise tools to manipulate organelles, chromosomes and microtubules [8]–[14]. Recently femtosecond laser pulses were demonstrated to cut single axons in vivo [15]–[18]. Despite the success of optical techniques in-vivo, this approach has rarely been used in in-vitro to explore axonal injury models that were otherwise inaccessible. Most in vitro axoprotection studies have been carried out by cutting axons with scalpels, needles, or glass pipettes [19]–[23], and only recently single-axon transection has been demonstrated using a nanoknife [24]. However all these mechanical approaches share the disadvantage of tearing and pulling transected membranes during the cut process and this would interfere with the regeneration ability of cells [16]. Ultrafast laser-based transections have the advantage of producing rapid clean cuts without tearing and pulling transected membranes. In addition, they are easy to automate [12], [14], [25], [26]. Recently, 180 ps visible (532 nm) laser pulses were used to cut axon bundles in vitro [27]. Hellman et al. showed that high-energy (600–800 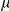
J) single pulses are enough to transect axon bundles in cultured dorsal root ganglion cells. These authors demonstrated that optical transection is also compatible with protein-patterned substrates, a useful technique for micro-manipulating the cell environment [28], [29]. Nanosecond pulses were also utilized to study the axon response to laser exposure [30], showing evidence of chemoattractant molecules secreted at the injury site. The growth cones of the injured cells, as well as the ones of adjacent cells turned and migrated toward the injury site.

Here we demonstrate that low energy picosecond laser pulses can achieve transection of unlabeled single axons *in vitro* with micron resolution. Using as a model a cell line (RGC-5) that when exposed to a low concentrations of staurosporine becomes differentiated and develops a neuronal morphology [31], [32], we established the conditions to achieve axon transection and characterized RGC-5 axon regeneration and cell body response to injury using time-lapse microscopy. Both isolated axons and cell bodies were imaged for more than 24 hr after injury. Using the platform we describe, we characterized the trajectories and mobility of injured cells and correlated them with the initiation of a regenerative response. As described by Wu et. al. [30], we also observed the secretion of chemoattractant molecules at the injury site and we found that RGC-5 cells responded to this signal, moving the somas toward the injury site. We also found that this chemoattractant signal is a function of the damage level of the cells.

Finally, we demonstrate how this platform can be used in high-content screening, cutting tens of cells per dish and automating the image acquisition and analysis of approximately 300 images per cell. By allowing calculation of kinematic parameters that describe the axonal and soma response to injury, this platform can be used for mechanistic studies in axonal injury and development of new axoprotective therapies.

## Results and Discussion

**Figure 1:**
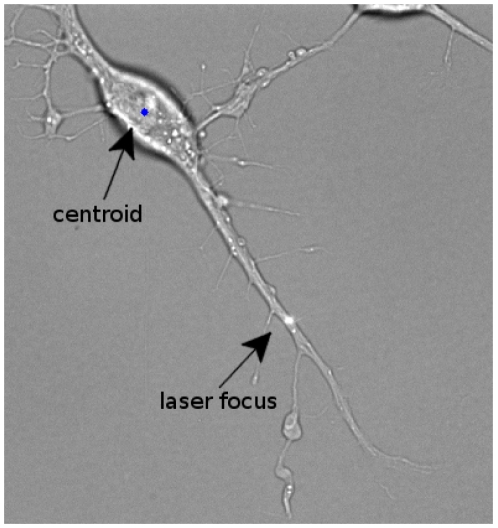
Axon transected with a focused picosecond laser beam. The pulse energy was 7.5 nJ at 76 MHz, with an exposure of 5 sec.

We coupled a picosecond laser, an inverted microscope with a high-resolution motorized stage, an environmental chamber, and custom-written image analysis tools to create a semi-automated platform for high-content study of the response of individual axons to transection. In a typical experiment, a culture dish of differentiated RGC-5 cells was placed on the microscope stage inside a compact incubator. The sample was then manually scanned to preselect axons to be injured and areas to be imaged. Subsequent steps were performed automatically under the control of custom software. Cell damage was sequentially induced by exposing axons to the focused laser beam at each selected position (Fig. 1). Cells were then sequentially imaged over up to 26 hr by positioning the motorized stage at each selected location, automatically adjusting the focus, normalizing the illumination intensity, and acquiring a transmission bright-field microscopy image. The stage was then moved to the following cell to repeat this sequence. The resultant time-lapse series allowed observation in parallel of the morphological evolution of RGC-5 axons and somas after axotomy under a variety of conditions.

**Figure 2:**
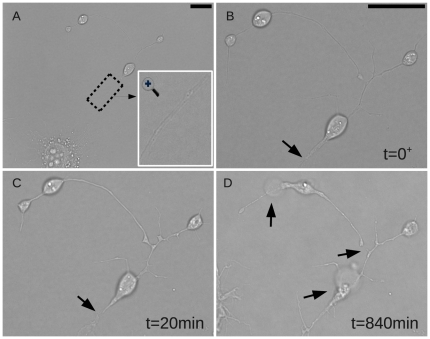
Longitudinal changes in axonal morphology after laser axotomy. A. Cell before injury and a magnification of the axon shown in an inset. B. After laser exposure there was swelling of the distal axon adjacent to the injury site. C. 20 min after exposure an approximately 1 m separation became apparent between the ends of the axon proximal and distal to the injury site. D. The loss of membrane integrity was evidenced by bubbles and distal axon fragmentation. Scale bars: 10 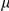
m. (See Supplemental Video S1 for the full series).

Parameters for laser axotomy were optimized in preliminary experiments to induce a focal axonal transection, without concomitant injury to other areas of the axon or soma. Specifically, the power of the laser beam was adjusted so that there was no immediate visible damage at the transection site but yet transection could be observed within several minutes. Typically this was achieved with exposure duration of 5 s and the pulse energy at the sample set at approximately 7 nJ (500 mW mean power). At these energy levels, RGC-5 axons were transected and yet the somas were able to re-extend their axons, i.e. axon transection was achieved while preserving the integrity of the proximal axon and soma (Fig. 2). Fig. 2A shows the whole cell and a magnified image of the axon to be injured. Immediately after laser injury (Fig. 2B), there was swelling of the distal axon adjacent to the injury site without visible transection. Within 20 min an approximately 1 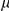
m separation became apparent between the ends of the axon proximal and distal to the injury site (Fig. 2C). The injury subsequently induced a degenerative process in the distal axon and a contraction in both ends of the isolated axon (Fig. 2C, D). The loss of membrane integrity was evidenced by bubbles and distal axon fragmentation (Fig. 2D). The entire image sequence is shown in Supplemental Video S1 including depiction of the soma trajectory as detected by our algorithm. The injury site is indicated trough the video by a blinking white dot.

The biological response to axonal injury differs among cells, presumably due to differences in the level and duration of induced damage, baseline susceptibility, environmental conditions, and interactions with adjacent cells. As an example of the power of this platform to study the neurobiology of axonal injury, we correlated two different aspects of the cellular response to injury. First, we distinguished three groups of RGC-5 cells based on morphological responses to axonal injury, that is whether they initially regenerated their transected axon. In group A (65% of total), cells either re-sealed the injured axonal membrane and hence reversed axon transection, reconnected the transected sections (Supplemental Video S2), or grew new processes at the damage site (Supplemental Video S3). In Supplemental Video S2 after a process was cut, the cell body began migrating toward the injury site, while at the same time the cut process started to degenerate, as evidenced by the retraction of its most distal part. This cell was then able to reconnect the transected process and degeneration stops. In Supplemental Video S3 there was a rapid axon degeneration after laser transection. The cell body moved toward the injury site and new processes grew at the same location as the laser transection site. We grouped these two kind of responses-reconnection of transected processes or regrowth of new processes at the injury site-together characterized by recovery or regeneration of RGC-5 cells after axonal injury.

In group B (35% of total), we include cells that neither repaired nor replaced their injured axons. This response is illustrated in the Supplemental Video S4 ,where after injury the transected process degenerated in less than 1 hour, but the cell body remained in the same position for approximately 500 min. After this latency the cell moved away from the injury site. We defined this behavior as a non-regenerative RGC-5 response to axonal injury. Finally in group C (5% of total), injured cells died within one day of injury. This response is illustrated in Supplemental Video S5.

Using this classification of RGC-5 cells response to injury, we measured cell motility after axotomy and found that it depended on the regenerative response and the initial axon length.

### Cell motility response

**Figure 3:**
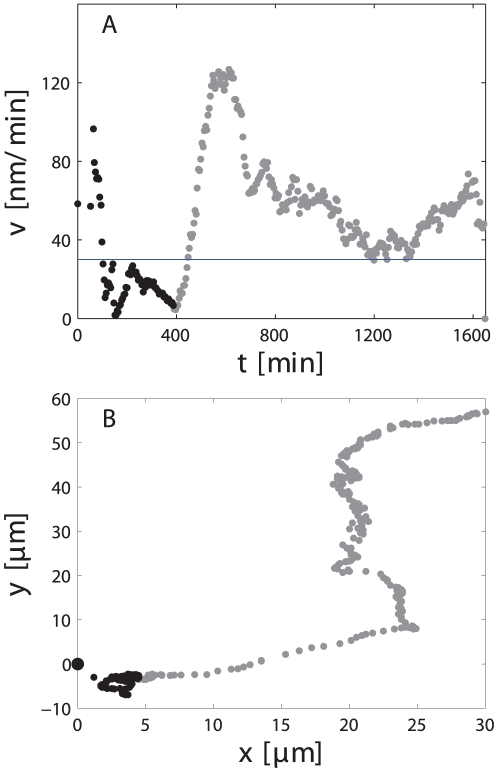
Soma motility response to axonal injury quantitative analysis. A. Instantaneous velocity and B. trajectory of the cell shown in Supplemental Video S4. Transection occurred at t = 0. The horizontal line in A shows the mean baseline velocity of control cells. Point were acquired every 5 minutes approximatelly.

To measure the motility of cell bodies after axotomy more than 300 images from each of the 40 axotomized cells and 9 control cells were processed using a custom-made algorithm. The position of the cell body over time was used to compute the trajectory and instantaneous velocity (see section on image processing). As an example, Fig. 3 depicts the instantaneous velocity and trajectory of the cell shown in Supplemental Video S4. The horizontal line represents the baseline mean velocity of 9 control cells (30 nm/min).

The change in motility in response to axotomy could by divided into an initial and a late response. A dramatic increase in cell motility was typically observed immediately after exposure (Fig. 3A). In this example the cell tripled its baseline velocity approaching 100 nm/min after transection of its axon. This increase in motility was followed by a latency phase, lasting approximately 6 hr, where the velocity returned to baseline. This latency phase was consistently followed by a sustained migration away from their initial position. Despite the initial motility increase, cells moved close to the initial position during this early response. This is shown in the trajectory plot (Fig. 3B), where the points that correspond to the first 450 min are shown in black and it can be seen that it represented only a small fraction of the entire trajectory. We characterized the initial response by the maximum velocity the cell reached within the first 150 min and called it reaction velocity (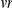). Moreover, we quantified the late response by computing the average velocity of the cell body during the time it velocity was higher than the basal velocity. That is, we did not compute the period of time in which cells were resting as illustrated in Supplemental video S4 and Fig. 3. After injury this cell remained in the same place for almost 500 minutes, after which it started to migrate away from the injury site. In this case the cell motility would be understimate if the velocity was averaged over the entire imaging session.

**Figure 4:**
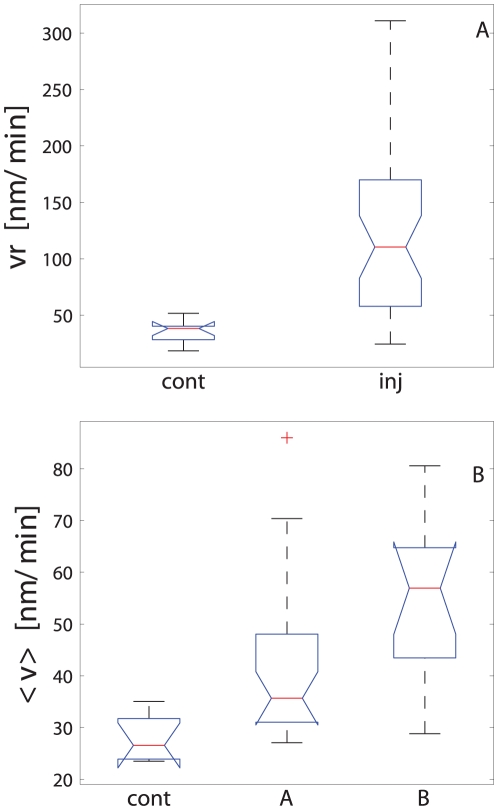
Soma motility response to axonal injury group comparisons. A. Initial reaction and B. long-term response. Cell motility changes were correlated to the regenerative response. Cell group labels are ***inj*** for injured cells, ***cont*** for control cells, ***A*** for that cells that either regrowth new axons or repaired injured one and, ***B*** represents cells that were not able either to regenerate or replace injured axons.

Data are presented using notched box plots [33], where the distribution median is represented with a red line and the upper and lower quartiles are the extremes of the blue boxes (see Fig. 4). The box notches are useful to illustrate significant differences between distributions. If notches do not overlap, the medians are significantly different at approximately 95% confidence. Data are considered outliers if they do not fall within a normal distribution, i.e. outside the interval defined as 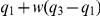 and 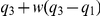, where 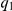 is the lower quartile, 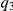 the upper quartile and 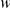 is called whisker. The parameter 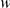 was set to 1.5, and assuming a normal distribution, this value leaves 99.3% of the data within that interval. The maximum and minimum measured values are connected with a dashed vertical line and outliers are represented by the + symbol. Protruded notches were used when the median was close to the upper or lower quartiles.

### RGC-5 soma motility and regenerative response

The initial motility response (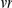) to axonal injury was significantly higher (125±77 nm/min vs. 35±10 nm/min; p = 0.008) than in control cells (Fig. 4A). Cells that regenerated or replaced their injured axons (group A) had an intermediate 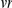 between non-regenerating axotomized cells(group B) and control cells (110±72 nm/min; p = 0.03 compared to control cells). The late motility response to injury followed the same pattern (Fig. 4B), with significantly higher velocities in the axotomized somas compared to controls (46±15 nm/min vs. 28±4 nm/min; p = 0.001). The late change in cell motility correlated with the regenerative response to injury: cells that regenerated their axons had a lower mean motility than cells that did not (54±15 nm/min vs. 42±14 nm/min; p = 0.015). These results demonstrate a correlation between the regenerative response of cells and their motility.

### The regenerative response of RGC-5 to axotomy correlates with axon length

**Figure 5:**
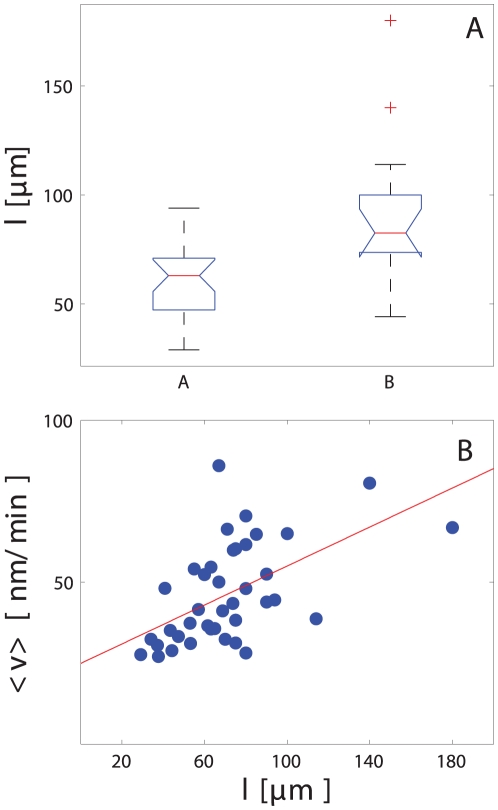
Effects of axon length on regeneration and motility. **A**. The regenerative response of cells depended on axon length. Cells with longer axons were less likely to reconnect or regrow their axons after axotomy. **B**. Correlation between cell motility and axon length. Cell with longer axons showed a higher motility after axotomy (R = 0.3 for linear regression).

Whether a cell regenerated its axon after axotomy was inversely dependent on the baseline length of the axon. The mean axon length of cells that regenerated their axons (group A) was 60±16 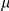
m, compared to 90±35 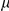
m for group B of cells that did not regenerate their axons (p = 0.0007); (Fig. 5A). These data imply that RGC-5 cells with longer axons were less likely to initiate a regenerative program after axotomy compared with those with shorter axons.

### RGC-5 soma motility is directly related to the length of the severed axon

The motility of axotomized RGC-5 somas was lesser when the axon regeneration program was initiated (Fig. 3). Furthermore, the likelihood of regeneration was greater when the axon length was shorter (Fig. 5). We therefore predicted that RGC-5 soma motility would be directly correlated with axon length, and this was indeed the case. In Fig. 5B the mean velocity is plotted as a function of the axon length, with cell motility increasing with greater axon lengths.

### RGC-5 cell migration correlates with the likelihood of regeneration

**Figure 6:**
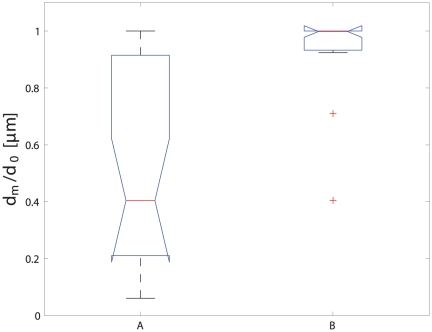
Cell motility after injury depends on whether an axon regeneration program is initiated. Somas that regenerated or repaired injured axons were more likely to migrate towards the site of axon transection (A), while somas that did not maintain their axons migrated away from the transection site (B).

A surprising observation that arose from analyzing multiple cells in parallel was that axotomized cells tended to migrate towards the injury site (see Supplemental Video S2 , S3 and S4). To quantify this phenomenon, we measured the minimum distance between the cell body and the injury site (d*_m_*) during the migration, and compared it to the initial distance d_0_. Cells that approached the injury site therefore tended to have the ratio d*_m_*/d_0_<1 (see Supplemental Video S2 and S3) and cells that moved away d*_m_*/d_0_ = 1 (see Supplemental Video S4). The migration of the soma towards the injury site also correlated with the regenerative response (Fig. 6), with cells that regenerated their axons being (A) much more likely to migrate toward the transection site (p = 0.0002). These results are in agreement with recent results [30] obtained with goldfish RGC cells, as we observed a migration of the whole cell to the injury site, presumably as a result of the secretion of specific molecules at the injury site. Our experiments show that both attraction and repulsion can be triggered depending to the level of damage.

### Conclusions

We demonstrated how a semi-automated picosecond laser-based platform for axonal injury can be used to study the biology of the axon response to transection. We established correlations between cell motility after injury, axon length, and the initiation of the regeneration response. RGC-5 cells with transected axons showed a change in motility as a response to injury, and this motility change correlated with the ability of RGC-5 cells to regenerate their transected axons (Fig. 4). Cells that were capable of repair or regrowth of the damaged axon migrated more slowly than cells that were not able to repair the damage. Moreover, we established that cells with long axons could barely recover their injured axons (Fig. 5A), and such cells showed a much higher motility (Fig. 5B). Afetr We also found that cells that either reconnected or regrew prAfetrocesses migrated towards the injury site, and cells that were not able to reconnect or regrow moved away from the injury site (Fig. 6). Taken together, these results suggest that RGC-5 cell motility was related to the level of injury and also that the liberation of chemoattractant molecules from the injury site depend on the injury level. It is tempting to speculate that the cell classification that was observed reflects the damage that was induced. Thus, RGC-5 cells that were slightly injured repaired their axon; RGC-5 cells that were more severely injured and were not able to reconnect their axons, replaced them with new processes; and cells that were dramatically injured could not self-repair even if the integrity of isolated axons was preserved. This analysis suggests that RGC-5 cells with long axons are less capable of reconnecting or replacing an injured process. This behavior could be explained by a macromolecule or organelle transport phenomena toward the injured axon, with longer neurites needing more time to be reconnected. However we also observed that long processes appear to survive longer than short processes, implying that this behavior cannot be simply explainded in terms of molecular transport toward the injury site and distal axon. Further experiment work is requiered to elucidate why shorter axons are more likely to be reconnected or replaced by injured cells.

Our results using the RGC-5 line are encouraging and demonstrate that laser-assisted transection can be used in primary cultures to help answer useful questions about axon protection and recovery after injury. The platform we described allowes the induction of highly controlled axonal damage with subcellular resolution and the the experiments demonstrate how low-energy picosecond pulses can be utilized for intracellular surgery. These lasers are useful to induce high-precision neuronal axotomy, avoiding collateral tissue damage, and yielding clean transection without pulling or tearing the axonal or somal membrane. Axon recovery and regeneration after injury was observed, showing that the damage induced by the laser was well localized.

This platform can be used for improving our understanding of the molecular mechanisms of the axon response to injury. It laso allows novel high-content screens for neuroprotective and axoprotective molecules which could potentially lead to therapies for diseases associated with axonal injury.

## Materials and Methods

### Cell Culture

RGC-5 cells were kindly provided by Neeraj Agarwal, Ph.D. [34] and cultured as previously described [31], [32]. Cells at passage 9 to 20 were inoculated in Dulbecco’s modified Eagle medium (DMEM) at 1000 cells/cm^2^ on poly-D-lysine-coated glass-bottom dishes. Cells were then incubated at 37°C in 5% CO_2_/air for 6 hours to allow cells to attach to the dish surface. Once cells were adherent, staurosporine (316 nM) was added to induce cell differentiation. After 4 hours, culture media was replaced by DMEM modified for long-term imaging by replacing the sodium bicarbonate with HEPES (10 mM). Dishes were then moved to the microscope for imaging and laser axotomy. See Supplemental Materials File S1 for details on reagent and media preparation.

### Microscope and laser injury platform

An inverted microscope (Olympus IX-71, Tokyo, Japan) equipped with a motorized two-axis stage (Thorlabs MAX201, Newton, NJ) was used for both laser axotomy and time-lapse imaging. To keep the sample at constant temperature (37°C), a PDMI-2 micro-incubator (Harvard Apparatus, Holliston, MA) connected to a TC-202A temperature controller (Harvard Apparatus, Holliston, MA) was mounted onto the motorized stage. The microscope was equipped with a stepper motor (Prior Scientific A500-H249, Rockland, MA) for automating image focusing. A CCD camera, (QImaging Retiga EXi 1394, Surrey, BC, Canada) was used for imaging. Software for automating laser injury and imaging was programmed using LabVIEW (National Instrument, Austin, TX). An autofocus system based on the intensity of the back-reflection of a secondary laser, was used to compensate for mechanical and thermal drifts during experiments lasting 24 hr or more.

### Axonal transection and imaging

Axonal transection and imaging were performed using the same microscope without removal of the sample. Dishes containing differentiated RGC-5 cells were placed in the microscope incubator and approximately 20–30 locations on the dish were manually selected by the operator for subsequent injury and imaging.

Cellular injury was induced by exposing the axon to the focused laser beam (pico-TRAIN High Q Laser, Rankweil, Austria). Fig. 1 shows an example of how cells were inured. The focus of the laser beam is inidcated. The pulse energy was varied between 5 nJ and 7.5 nJ while the exposure duration was set to 5 sec. The wavelength of the laser was 1.064 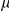
m and the pulse width was 6ps. Under these exposure conditions, cell damage was not always immediately visible after exposure, but axon transection and degeneration became evident with time (see Fig. 2). Details of the microscopy set-up and alignment protocols are further described in the Supplemental Materials File S1 and also in Fig. S1, where a schematic of the microscope is presented.

After injury, the morphological evolution of axons and somas was imaged by time-lapse transmission bright field microscopy over up to 26 hours, by positioning the motorized stage to each previously selected location. Once in position, the program adjusted the focus and illumination intensity, acquired an image, moved the stage to the following cell and repeated the sequence. The time between sequential images was 10–20 sec, depending on the distance between cells, and each cell was imaged approximately 300 times with a duty cycle of 5 to 10 minutes, depending on the total number of cells imaged and the distance between them. The cell density was kept low to avoid overlap of axons. Therefore in order to image enough cells, a relatively large area (approximately 15 mm^2^) had to be scanned.

### Image Processing

**Figure 7:**
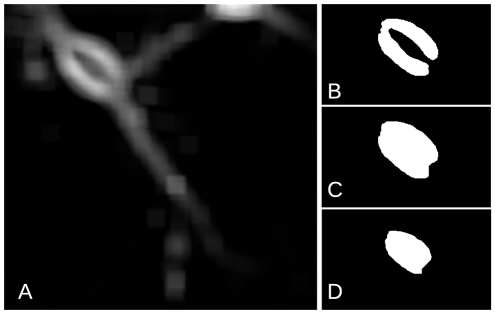
Intermediate steps in the image analysis algorithm. **A**. Standard deviation of the image intensity over a 90-pixel matrix. **B**. Thresholding. **C**. Opening and filling morphological operations. **D**. Erosion yielding the final mask.

Approximately 300 images could be acquired at each of up to 50 locations, making up to 15,000 images per experiment. We developed an algorithm in MATLAB (MathWorks, Natick, MA) to determine the movement of somas after injury. The algorithm has two parts: detection and tracking. Somas were detected using the process depicted in Fig. 7 in order to identify the positions of each cell in all image frames. The standard deviation of the intensity in square matrices of 90 pixels (Fig. 7A) was thresholded and a binary image obtained (Fig. 7B). A mask was computed by closing and filling (Fig.7C), and finally eroding (Fig. 7D) the binary image. The centroids of all objects obtained in the resultant mask were used to assign a position to the somas present in each frame (see Fig. 6 for an example). The positions obtained were then processed with a single particle-tracking algorithm to calculate the cell trajectories [35] (See supplemental material File S1 for the complete code).

## Supporting Information

Video S1 Longitudinal change in axonal morphology after laser axotomy. After exposure the distal part of the axon start a degeneration program evidenced by the retraction of both ends. The loss of axonal membrane integrity is evidenced by bubbles formation and axon fragmentation.
(AVI)

Video S2 Reconnection of transected process. After transection the isolated process started to degenerated. The degeneration is interrupted after the cell reconnected the transected process.
(AVI)

Video S3 Growth of new process. Some cells were not able to reconnect their transected processes and instead grew new processes to the injury site.
(AVI)

Video S4 Some cells neither repaired nor replaced the injured axon. These cells migrated away from the injury site.
(AVI)

Video S5 5% of the injured cells died within one day of injury.
(AVI)

Figure S1 Microscope Set-up.
(EPS)

File S1 Supporting material.
(PDF)
