# Supplementary material for: Laser-Based Single-Axon Transection for High-Content Axon Injury and Regeneration Studies
Source: PLoS One. 2011 Nov 2;6(11):e26832. doi: 10.1371/journal.pone.0026832 (PMC3206876; doi:10.1371/journal.pone.0026832)
Supplement: File S1 — Supporting material. (PDF) [file pone.0026832.s007.pdf]

Supplemental Materials

Reagents and media preparation

Sodium Piruvate 100mM, HEPES 1M, L-Glutamine MW 146.15, Dulbeccos modified Eagles medium (DMEM) with 4.5g/L Glucose, with L-glutamine and, DMEM (powder) without Sodium Bicarbonate, without Phenol Red, without L-glutamine, without Sodium Piruvate with 4.5g/L Glucose were from WISENT Inc. (Quebec, Canada). Staurosporine was from Alexis Biochemical (San Diego, CA). The media filter system (0.22μm Polyesther-sulfone membrane) was from CORNING (New York, USA). 35mm Optical culture dishes coated with Poly-d-lysine were from Mattek (Massachusetts, USA).

Regular DMEM was supplemented with 10% Fetal Bobine Serum (FBS) and 100 U/mL penicillin and 100 μg/mL streptomycin. Imaging air running DMEM without sodium bicarbonate was prepared from powder as described by vendor and supplemented with 10% FBS, 100 U/mL penicillin and 100 μg/mL streptomycin, Sodium Piruvate (1mM final concentration), L-Glutamine (final concentration 584mg/ml) and HEPES (10mM final concentration). The pH was adjusted between 7.2-7.4 adding Sodium Hydroxide 10N and the media was filtered through a 0.22μm membrane.

Axotomy Set-Up

In Fig. S1 a scheme of the microscope is shown. The laser beam (picoTRAIN High Q Laser, Rankweil, Austria) was coupled to the microscope through a lateral port and directed to the objective with a 70-30% reflection-transmission beam-splitter. In this way we were able to acquire transmission images at the same time we illuminated the sample with the laser. The power of the laser was adjusted using a half wavelength plate and a polarizer. The size of the laser beam was resized to overfill the back aperture of the objective with a two-lens telescope.

To focus the laser beam at exactly the object plane of the microscope, we adjusted the distance between the telescope lenses as follows: a sample made of silica beads of 0.9μm adsorbed to a cover slip was imaged with the camera in transmission mode (see bead adsorption protocol below). With the laser turned on, the back-reflection from the glass-water interface was observed in the beads image and the distance between the telescope lenses was adjusted

to minimize the size of such laser spot in the image.

Beads coated dishes

Silica 0.5-0.9um micro-spheres were purchased at Bang Laboratories (Indiana, USA). To allow beads adsorption to the optical dish, a dilution 1:200 in deionized water was incubated 30-45 minutes.

Code Description

1) Mask Creation. From the transmission Images we create first binaries files with the soma of each cell.

2)Once Mask were created we keep the position of the cell's soma as the centroid of each cell detected by the binarizartion process.

3) Trajectories reconstruction from the detected soma's position.

```
samplePath='path to the image files';

n=90;
n2=15;
r=0.6;
msk = ones(n);
se = strel('disk',n2);
        imagesList=dir([samplePath '*.tif']);
        mkdir([maskPath thisSample]);
        thisMask=[maskPath thisSample '/'];
        N=length(imagesList);

        for j=1:N
        m=imread([samplePath imagesList(j).name]);
% calculation of the standard deviation over the image using as a
%windows the msk matrix stdfilt is a matlab function.
        Y = stdfilt(m,msk);
% binarization of the image
        maximum=max(max(Y));
        minimum=min(min(Y));
        level=r*(maximum-minimum)+minimum;
        bwImage=(Y>=level);
% Morphological modification of the image
        mask=imclose(bwImage,se);
        mask=imfill(mask,'holes');
        [mask, numObjects]=discardSmallObjects(mask,0.3);
        mask=im2bw(mask,0.5);
   seD = strel('diamond',20);
        somaMask = imerode(mask,seD);
        mask=im2bw(mask,0.5);
        imwrite(mask,[thisMask 'mask' imagesList(j).name],'tif','compression','none');

   %keyboard
        end
```

2) Get the position of the centroide of detected somas.

```
        ==============================================================
        listMask=dir([thisSampleMaskFolder '*.tif']);
        N=length(listMask);
        positions=[];
        for i=1:N;
        thisMask=imread([thisSampleMaskFolder listMask(i).name]);
```

```
        thisMaskPositions=getSomaCentroids(thisMask);
        thisMaskPositions=[thisMaskPositions ones(size(thisMaskPositions,1),1)*i];
        positions=[positions; thisMaskPositions];
        end
        positionsPath=[thisSampleResultsFolder 'positions.dat'];
        fid=fopen(positionsPath,'w');
        fprintf(fid, '%5.3f\t %5.3f\t %d \r\n',positions');
        fclose(fid);
        end
================================================================
function [positions]=getSomaCentroids(mySomaMask)
[somaLabeled, numObjects]=bwlabel(mySomaMask, 8);
somaData=regionprops(somaLabeled, 'basic');
positions=zeros(numObjects, 2);
        for itObject=1:numObjects
                positions(itObject, 1)=round(somaData(itObject,1).Centroid(1));
                positions(itObject, 2)=round(somaData(itObject,1).Centroid(2));
        end
================================================================
```

3) Trajectories reconstruction

```
listSamples=dir(resultsPath);
N1=size(listSamples,1);
%% set parameters to call the function track
maxDisplacement=100; %
lostFrames=10; % number of frames a particle can be lost
dimensions=2;
minLenght=5; % minimanl trajectory length in units of frames
quiet=0;

trackingParameters.mem=lostFrames;
trackingParameters.dim=dimensions;
trackingParameters.good=minLenght;
trackingParameters.quiet=quiet;
%%
for i=3:N1
        positionsPath=[resultsPath listSamples(i).name '/' 'positions.dat'];
        positions=importdata(positionsPath);
        trackResult=track(positions, maxDisplacement, trackingParameters);
        trackPath=[resultsPath listSamples(i).name '/' 'track.dat'];
        %'escribe'
        fid=fopen(trackPath,'w');
        fprintf(fid, '%5.3f\t %5.3f\t %d\t %d \r\n',trackResult');
        fclose(fid);
end
```

```
=============================================================================
%	track function was obtained from
%	http://physics.nyu.edu/grierlab/software/track.pro
%	[35]This code 'track.pro' is copyright 1999, by John C. Crocker.
%	It should be considered 'freeware'- and may be distributed freely
%	(outside of the military-industrial complex) in its original form
%	when properly attributed.
```
